# Comparing the effectiveness of an enhanced MOtiVational intErviewing InTervention (MOVE IT) with usual care for reducing cardiovascular risk in high risk subjects: study protocol for a randomised controlled trial

**DOI:** 10.1186/s13063-015-0593-5

**Published:** 2015-03-25

**Authors:** Adam Bayley, Nicole de Zoysa, Derek G Cook, Peter H Whincup, Daniel Stahl, Katherine Twist, Katie Ridge, Paul McCrone, Janet Treasure, Mark Ashworth, Anne Greenough, Clare Blythe, Kirsty Winkley, Khalida Ismail

**Affiliations:** Department of Psychological Medicine, Institute of Psychiatry, King’s College London, 10 Cutcombe Road, London, SE5 9RJ UK; Population Health Research Institute, St George’s, University of London, Cranmer Terrace, London, SW17 0RE UK; Department of Biostatistics, Institute of Psychiatry, King’s College London, 16 De Crespigny Park, London, SE5 8AF UK; Department of Health Services and Population Research, Institute of Psychiatry, King’s College London, 16 De Crespigny Park, London, SE5 8AF UK; Section of Eating Disorders, Institute of Psychiatry, King’s College London, 16 De Crespigny Park, London, SE5 8AF UK; Department of Primary Care and Public Health Sciences, King’s College London, 42 Weston Street, London, SE1 3QD UK; Division of Asthma, Allergy and Lung Biology, King’s College London, Guy’s Hospital, London, SE1 9RT UK

**Keywords:** Cardiovascular disease, Physical activity, Diet, Accelerometer, Motivational interviewing, CBT, Primary care, Health trainers

## Abstract

**Background:**

Interventions targeting multiple risk factors for cardiovascular disease (CVD), including poor diet and physical inactivity, are more effective than interventions targeting a single risk factor. A motivational interviewing (MI) intervention can provide modest dietary improvements and physical activity increases, while adding cognitive behaviour therapy (CBT) skills may enhance the effects of MI. We designed a randomised controlled trial (RCT) to examine whether specific behaviour change techniques integrating MI and CBT result in favourable changes in weight and physical activity in those at high risk of CVD. A group and individual intervention will be compared to usual care. A group intervention offers potential benefits from social support and may be more cost effective.

**Methods/Design:**

Individuals aged between 40 and 74 years in 11 South London Clinical Commissioning Groups who are at high risk of developing CVD (≥20%) in the next 10 years will be recruited. A sample of 1,704 participants will be randomised to receive the enhanced MI intervention, delivered by trained healthy lifestyle facilitators (HLFs), in group or individual formats, in 10 sessions (plus an introductory session) over one year, or usual care. Randomisation will be conducted by King’s College London Clinical Trials Unit and researchers collecting outcome data will be blinded to treatment allocation. At 12-month and 24-month follow-up assessments, primary outcomes will be change in weight and physical activity (average steps per day). Secondary outcomes include changes in low-density lipoprotein cholesterol and CVD risk score. Incidence of CVD events since baseline will be recorded. A process evaluation will be conducted to evaluate factors which impact on delivery, adherence and outcome. An economic evaluation will estimate relative cost-effectiveness of each type of intervention delivery.

**Discussion:**

This RCT assesses the effectiveness of a healthy lifestyle intervention for people at high risk of CVD. Benefits of the study include the ethnic and socioeconomic diversity of the study population and that, via social support within the group setting and long-term follow-up period, the intervention offers the potential to support maintenance of a healthy lifestyle.

**Trial registration:**

This trial is registered with the ISRCTN registry (identifier: ISRCTN84864870, registered 15 May 2012).

## Background

### Epidemiology of cardiovascular disease and its risk factors

Cardiovascular disease (CVD) is the most common cause of death (and premature death), morbidity and disability in middle-aged and older people both in the United Kingdom and in other developed countries [1]. However, CVD is highly preventable as many of the major determinants of CVD are modifiable, including cigarette smoking, a diet high in saturated fat, high serum cholesterol, obesity, sedentary lifestyle, hypertension and diabetes [1-5]. The risk of CVD varies markedly between ethnic groups, with a higher rate of ischaemic heart disease in South Asians and a higher rate of cerebrovascular disease in Africans amongst those living in England and Wales [6].

Although CVD remains the most common cause of death in developed nations, mortality rates have been falling. Between 1981 and 2000, CVD mortality in the United Kingdom fell by 62% in men and by 45% in women [7]. Cohort studies [8] and prediction models [7] suggested that a fall in the prevalence of cigarette smoking, a decline in population blood pressure levels and changes in cholesterol levels were important contributors. Population-wide changes in modifiable risk factors can bring about substantial benefits and further changes in blood lipids, particularly non-high density lipoprotein (HDL) cholesterol, could be achieved through population-wide dietary changes [8]. However, limited changes in physical activity and rising levels of obesity have limited the decline in CVD mortality [8]. Further efforts are therefore needed to bring about positive changes, particularly in diet, obesity and physical activity.

### The evidence for dietary interventions

Systematic reviews of randomised controlled trials (RCTs) of generic dietary advice interventions for reducing CVD risk in the primary prevention setting have generally found small beneficial effects on mean total and low-density lipoprotein (LDL) cholesterol levels, as well as small reductions in blood pressure, but HDL cholesterol and triglyceride levels remain unchanged [9]. Most studies were conducted in the United States, with a short average duration of 10 months. Compared with usual care, dietary instruction interventions produce modest weight losses and these diminish over time [10].

### The evidence for increasing physical activity

Physical inactivity increases overall mortality and the risk of many diseases, including CVD and diabetes [11]. The Department of Health advises adults to perform at least 30 minutes of at least moderate intensity physical activity on five or more days per week, in at least 10 minute bouts, for optimum health benefits [11]. Walking is the most common form of physical activity in adults and is promoted as a near perfect exercise as it has the lowest risk of harm, and is now public health policy in the United Kingdom [12,13].

However, the proportion of those achieving these recommendations is low, particularly when objective measures are used to assess physical activity. In England, 39% of men and 29% of women self-report achieving the recommended physical activity levels, but objective assessment of physical activity using accelerometers in a sub-sample of the Health Survey for England found that only 5% of men and 4% of women aged 35 to 64 years achieved the recommended levels [14]. A Cochrane review of 17 RCTs reported moderate positive short-term increases in physical activity following health interventions, either in a group or individual format, but findings were limited since most studies used self-report measures in motivated volunteers [15]. There is mounting evidence that the use of pedometers as a method of aiding self-monitoring can increase physical activity and improve health in the short term [16]. Social support and cognitive behaviour therapy (CBT) strategies, rather than health education alone, are now recommended in older adults [17].

### The evidence for motivational interviewing

The Cochrane Heart Group systematic review of multiple risk factor interventions observed that techniques based on instruction and information such as workshops, lectures, provision of written material, assignments, shopping tours and cooking sessions were associated with small improvements in lipid levels and reductions in blood pressure, especially when embedded in a theoretical framework related to behaviour change [18]. Motivational interviewing (MI) is a common approach to behavior change in health care defined as a collaborative, goal-oriented style of communication with particular emphasis on the language of change [19]. It is designed to strengthen personal motivation for, and commitment to, a specific goal by eliciting and exploring the person’s own reasons for change within an atmosphere of acceptance and compassion [20]. An MI intervention moves through the following processes: engaging, focusing, evoking and planning. The core skills of MI can by summarised by the acronym OARS: Open question, Affirmations, Reflections and Summaries. The appeal of MI is that it is brief, can be delivered by a range of health providers and has a competency framework. Systematic reviews and meta-analyses have consistently shown that MI techniques have a moderate effect on diet and exercise (effect sizes (d) of 0.53 standard deviations in four RCTs) [21]. In another meta-analysis of MI interventions, significant effects were seen for weight reduction and for reducing cholesterol, although the number of trials was few [22].

The effects of MI can be short-lived. We found that four sessions of MI alone was not associated with improved glycaemic control in people with type 1 diabetes, but four sessions of MI followed by eight sessions of CBT was associated with improved glycaemic control (compared to usual care [23]). These effects disappeared after 12 months [24]. During the process evaluation, it was found that MI helped people to become more ready to change their behaviours, but the change was less likely to be implemented without additional support [25]. However, the evidence for enhancing MI with CBT is not consistent as the landmark Combined Pharmacotherapies and Behavioral Interventions for Alcohol Dependence study (COMBINE) did not demonstrate increased abstinence in those receiving the psychological intervention [26].

### A taxonomy of behaviour change techniques

The epidemic of modifiable risk factors for CVD and the limitations of current models of lifestyle interventions, particularly their short-term effects, is leading to a search for more sophisticated and targeted behavioural interventions [27]. For instance, systematic reviews have shown that the components of behavioural interventions that appeared to be most effective in improving diet and physical activity were based on self-regulatory behaviours such as goal setting, self-monitoring, giving feedback, utilising social support and MI. Interventions based on a psychological theory (such as the theory of planned behaviour [28]) were more effective, as were those for high risk populations. There is less evidence to support a case for any minimum threshold of intensity, mode of delivery, intervention provider and setting [18,27,29]. Strategies to prevent relapses and to increase the maintenance of healthier lifestyles over longer periods remain poorly understood and understudied. Evaluating interventions in the context of a taxonomy of behaviour change techniques and an intervention map offer a framework that is easier to teach, test, replicate and translate [28-30].

### Cardiovascular risk

The NHS Health Check programme is part of the Department of Health’s long-term vision for the future of public health in England [31]. In offering checks to all those aged between 40 and 74 years without a known diagnosis of CVD the programme aims to prevent heart disease, stroke, diabetes and kidney disease and to reduce health inequalities. The risk assessment includes collection of demographic data, family history, smoking status, cholesterol, blood pressure and a diabetes filter using computerised risk engines such as QRISK, QRISK2 or the Framingham. An individualised management plan is then given according to the risk assessment to support lifestyle changes such as referral to smoking cessation services, exercise prescriptions, lifestyle advice and signposting to local resources. However, the programme has attracted criticism due to the lack of an up-to-date evidence base for the implementation of Health Checks, and Clinical Commissioning Groups (CCGs) lacking the resources to implement them [32]. Therefore, a wider evidence base is required to substantiate the benefits of a Health Check programme. In this trial, we aim to evaluate whether MI integrated with CBT can effectively prevent development of disease in those at high risk of CVD.

### The role of health trainers and healthy lifestyle facilitators

We propose to structure our intervention, which is based on MI integrated with CBT, around health trainers. The deployment of health trainers, or healthy lifestyle facilitators (HLFs), into the public health workforce has the potential to address health inequalities; an important issue within the multi-ethnic and variably deprived South London boroughs. They are usually drawn from the local community they serve and are trained in a variety of settings with national accreditation. Their role includes identifying clients from hard-to-reach disadvantaged groups, working one-to-one to assess lifestyle and wellbeing, identifying problem areas, setting goals, supporting behaviour change and reviewing their clients’ progress [33]. The potential for a HLF to deliver more sophisticated interventions, either in an individual or a group format, has yet to be studied [34].

### Summary

The potential benefits at the population level of modifying diets, reducing weight, reducing cholesterol levels and increasing physical activity are considerable. However, identifying the most effective intervention targeting lifestyle changes remains a challenge for researchers and policy makers. MI is an intervention that has broad appeal for its collaborative patient-centred style, brevity, evidence base and deliverability. The effects of MI could be enhanced by embedding it into a taxonomy of specific health behaviour change techniques, such as setting personal goals, offering physical tools to self-monitor (such as pedometers) and offering guidance and feedback. The relative effectiveness of a group versus an individual intervention remains uncertain, but the former offers ‘automatic’ social support and may be more cost-effective.

We propose to compare the effectiveness of 10 pre-specified behaviour change techniques (integrating MI with CBT and underpinned by the theory of planned behaviour and social cognitive theory) in reducing weight and increasing physical activity in those at high risk of CVD over 24 months in two formats, group or individual, with usual care.

## Methods/Design

### Design

**Figure 1:**
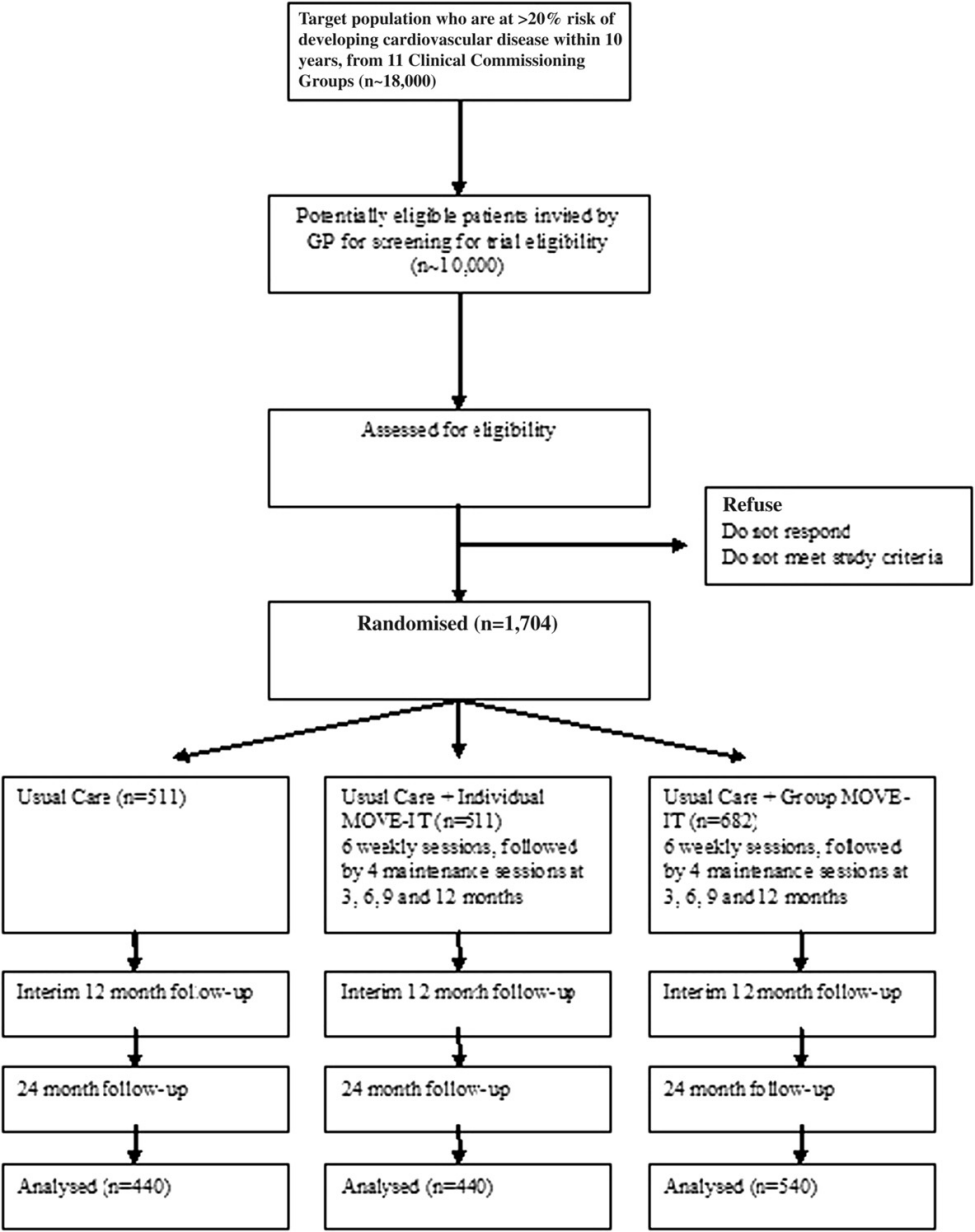
**Study flow chart showing progression through trial.** A 20% dropout rate is assumed.

This is a three-arm, parallel, multicentre RCT for people identified as at high risk for CVD. The three arms are usual care, usual care and enhanced MI in a group format and usual care and enhanced MI in an individual format. As participants of the group arm, but not the other two arms, are clustered within groups we have a partially clustered (or nested) design. Simple randomisation will be used, with GP practice included as a random factor in the model, and emphasis being on more practices and fewer patients per practice. The trial is funded by the National Institute for Health Research - Health Technology Assessment Programme (NIHR-HTA) and has been registered with the International Standard Randomised Controlled Trial Number (ISRCTN) registry (identifier: ISRCTN84864870). The study flow chart in Figure 1 shows progression through the study for individual participants.

### Primary objectives

#### Primary hypotheses

Enhanced MI, delivered in a group or individual format, is more effective than usual care in reducing weight and increasing physical activity (average number of steps per day assessed via accelerometry) 24 months later.

#### Secondary hypotheses

1. Enhanced MI delivered in a group format is more effective than in an individual format in reducing weight and increasing physical activity (average number of steps per day assessed via accelerometry) 24 months later.
2. Enhanced MI, delivered in a group or individual format, is more effective than usual care in reducing LDL cholesterol, reducing CVD risk score and increasing moderate to vigorous physical activity ((MVPA) amount of at least moderate physical activity in longer than 10 minute bouts) 24 months later.
3. Enhanced MI, delivered in a group or individual format, is more cost-effective than usual care, in terms of quality-adjusted life years (QALYs) gained over the 24 month follow-up period.

### Secondary objectives: process evaluation

#### Mediational analysis

We will conduct a mediational analysis to examine whether changes in behavioural and psychological factors such as dietary intake, health beliefs, depressive symptoms and self-efficacy mediate the association between the intervention and outcomes.

#### Effect modification and subgroup analyses

Subgroup analyses will be used to examine whether the association between the intervention and the outcomes are modified by sociodemographic, lifestyle and psychological factors measured at baseline, such as age, gender, ethnicity, family history of CVD, smoking status, alcohol intake, index of deprivation and depressive symptoms. The power for identifying significant effect modification will be limited.

#### Fidelity

A fidelity analysis (using mixed methods) will be conducted to assess whether the enhanced MI intervention is delivered according to the manual and to compare whether the level of competencies between the HLFs is associated with variations in outcomes using rating scales and thematic contents analysis of sessions.

#### Patient and therapist experience analysis

Using qualitative methods, we will describe the perceived expectations, benefits, strengths and limitations of the intervention from the patient and therapist perspective.

### Setting

The study is set in 11 South London CCGs (Bexley, Bromley, Croydon, Greenwich, Kingston, Lambeth, Lewisham, Richmond and Twickenham, Southwark, Sutton and Merton and Wandsworth) that are linked to each other by the South London Health Innovation and Education Cluster (SLHIEC), and inherent in this infrastructure is an efficient method for recruitment. South London has additional advantages: the population is nearly three million residents; nearly a quarter of the population is either African, Caribbean or South Asian; it spans the range of population densities, urbanisation and socioeconomic profiling; the development of a Health Innovation Network (HIN) in South London will allow rapid dissemination of research findings and it will be cheaper than a multicentre study across the United Kingdom, as research resources can be shared across adjacent CCGs during periods of varying workload. GP practices with list sizes greater than 5,000 patients will be invited to take part, representing approximately 60% of all practices within the SLHIEC. To recruit patients from every practice in the SLHIEC is not cost-beneficial, as smaller practices will have fewer patients to recruit from. A limitation is that the South London sample may not be representative of the rest of the United Kingdom, and not address the north and south regional health inequalities. However, there are many pockets of health inequalities within South London that mirror the rest of the United Kingdom, and we are well-placed in being in a geographical setting that has a significant proportion of Africans, Caribbeans and South Asians to address ethnicity in the design.

### Target population

The sampling frame will be GP practices with list sizes greater than 5,000 patients. The case definition includes adults aged between 40 and 74 years who screen as positive for high CVD risk, and not known to have CVD or to be on the diabetes, kidney, atrial fibrillation or stroke register. Following screening via the patient records database, the GP will invite those who are potentially eligible to participate in the study.

Informed consent is gained from all participants prior to undergoing screening in order to validate their eligibility to participate. High CVD risk will be calculated using QRISK2 (QResearch, Nottingham, UK), a validated predictive tool for identifying those at a 20% or higher chance of having a fatal or non-fatal cardiovascular event over the next 10 years [35]. The measures required for the calculation of QRISK2 score are age (years), smoking status, self-assigned ethnicity, systolic blood pressure, ratio of total serum cholesterol to HDL cholesterol, body mass index (BMI), family history of coronary heart disease in first degree relative, Townsend deprivation score, treated hypertension and diagnosis of rheumatoid arthritis.

### Criteria for including and excluding patients

The inclusion criteria are: fluent in conversational English, permanent residents and planning to stay in the United Kingdom for at least three quarters of the year and at high cardiovascular risk according to GP records (QRISK2 score ≥20%).

The exclusion criteria are: established CVD (including congenital heart disease, angina, myocardial infarction, coronary revascularisation procedures, peripheral artery disease, coronary artery bypass graft or angioplasty); having a pacemaker; on a register for diabetes, kidney disease, atrial fibrillation or stroke (either ischemic or haemorrhagic, including transient ischemic attacks); chronic obstructive pulmonary disease; disabling neurological disorder; severe mental illness such as psychosis, learning disability, dementia and cognitive impairment; registered blind; housebound or resident in nursing home; unable to move about independently or not ambulatory; more than three falls in past year; pregnancy; advanced cancer; morbid obesity (BMI ≥50 kg/m^2^); and current participation in a weight loss programme. When in doubt we will seek the GP opinion and approval.

### Sample size

The power calculation of our main outcome variables are based on the findings of previous research [16,36]. We have selected a very conservative effect size of 0.25 expressed as the difference in units of pooled standard deviations, which translates to an ability to detect a difference between two groups of 675 steps per day (physical activity), 1.25 kg weight and 0.25 umol/l total cholesterol at 24 month follow-up. Our study is powered to detect changes which may be modest at the individual level, but would have an important impact if occurring at the population level [37].

We took into account clustering effect within the group intervention (intraclass correlation coefficient = 0.05) by calculating the optimal sample size in presence of differential clustering effects [38]. A sample size of 1,420 participants in total are needed to detect these differences in our primary hypotheses, and a two-tailed alpha of 0.025 to take account of multiple comparisons of the contrasts ‘individual intervention versus control group’, ‘group intervention versus control group’ and ‘group versus individual intervention’. Assuming an approximate dropout rate of 20%, a total sample size of 1,704 (540 in group and 440 in individual and usual care) is needed.

### Baseline data

#### Sociodemographic data

Data on age, gender, self-report ethnicity, occupational status, educational attainment, marital status, literacy and family history of CVD will be collected.

#### Biomedical data

We will collect data on weight, height, BMI, waist circumference, lipids and glycated haemoglobin (HbA1c). We are not measuring fasting glucose as it is not essential to the current diagnosis criteria for diabetes. Weight will be measured in light clothing, without shoes, on a Class 3 Tanita SC240 weighing digital scale (Tanita, Tokyo, Japan) to 0.01 kg for weight and body fat composition. Height will be measured to 0.1 cm using stadiometers (Tantita, Tokyo, Japan) with the supported stretch stature method. Weight and height measurements will be used to calculate BMI (weight/height, kg/m^2^). Waist circumference will be measured horizontally halfway between the lowest rib and the upper prominence of the pelvis using a non-extensible steel tape against the bare abdomen. Blood pressure and resting heart rate will be measured with digital Omron BP monitors (Omron, Kyoto, Japan) using standardised procedures of the average of two readings taken one minute apart while seated. The QRISK2 score will be the research measure of CVD risk.

#### Lifestyle data

We will collect data on smoking status: if current how many cigarettes per day, ex-smoker (for how many years) and never smoked. We will collect and store blood samples for later measurement of cotinine levels. Alcohol intake will be measured using the Alcohol Use Disorders Identification Test [39].

Physical activity will be measured objectively using the ActiGraph GT3X accelerometer (ActiGraph, Florida, United States), a tri-axial movement sensor which also records step counts. The ActiGraph instrument has been validated and will collect data on number of steps taken and physical activity from sedentary to very vigorous [40]. The researcher will explain to the participant how to wear the accelerometer; on a belt over the hip for seven days, from waking in the morning until going to bed at night, and only removing for bathing. Participants are asked to keep a log of activities, including sedentary ones, to assist with the qualitative interpretation of the data. The output from the accelerometer includes number of steps and time spent doing physical activity using standard cut-off points for sedentary, light, moderate, vigorous and very vigorous physical activity. The researcher will ensure that, on the participant returning the accelerometer, it has been worn for at least 540 minutes on each of at least five days, and if not the participant will be asked to wear the accelerometer for another seven days. A measurement of physical activity at moderate level or greater (MVPA) in longer than 10 minute bouts will be extracted from the data collected.

Dietary intake will also be assessed. A standardised multiple-pass 24-hour dietary recall will be carried out as it can be more objective and more reliable as a measure of change in intervention studies. Researchers will be trained to follow a standardised protocol, ask neutral probing questions to encourage recall of food items, and taught about different methods of food preparations and brands in different cultures. Portion size will be assessed with food photographs to estimate daily calorie intake [41,42]. Total and non-HDL cholesterol will be measured as a proxy biomarker of change in dietary fat intake.

#### Psychological data

Health beliefs about diet, exercise and perceptions of risk for developing CVD and related conditions will be measured by the Brief Illness Perception Questionnaire, adapted for perception of risk [43]. Self-report physical activity will be measured by the Global Physical Activity Questionnaire (GPAQ) and International Physical Activity Questionnaire (IPAQ) [44]. Self-efficacy measures for physical activity and dietary habits will be included as psychological processes we are seeking to change during the intervention [45]. Depressive symptoms will be measured using the nine-item Patient Health Questionnaire [46], as depression is associated with worse outcomes in CVD [47].

### Randomisation and allocation concealment

Randomisation of participants will be conducted by the data manager from an independent Clinical Trials Unit (King's College London) using computer-generated randomisation blocks with block sizes of 10. In each block, 10 subjects will be randomised to group, individual or usual care in a 4:3:3 ratio. The unequal allocation ratio ensures that the group arm will have approximately 33% more patients, allowing the group sessions to run with a sufficient number of participants. As this is a complex intervention, it is not possible to conceal the allocation to the participants or the HLFs. Assessors and technicians will be blind to the allocation for the primary and secondary outcomes. There is a small inevitable risk that allocation will be revealed to the outcome assessors, which will be minimised by requesting and reminding participants not to reveal their allocation.

### Planned interventions

#### Group one: usual care

GPs participating in the study will be expected to follow their local Health Check pathway for those who have a CVD risk score of ≥20%.

#### Group two: usual care and enhanced motivational interviewing in a group format

##### Theoretical framework

The intervention will be based on the theory of planned behaviour [28] for initiation of behaviour change, which states that in order to change behaviour, people need to form an intention. Intention formation is influenced by three constructs: expected value or positive attitude (people see the value in making the change); subjective norm (significant others and peers also value the change) and self-efficacy (people believe they are capable of making the change).

**Figure 2:**
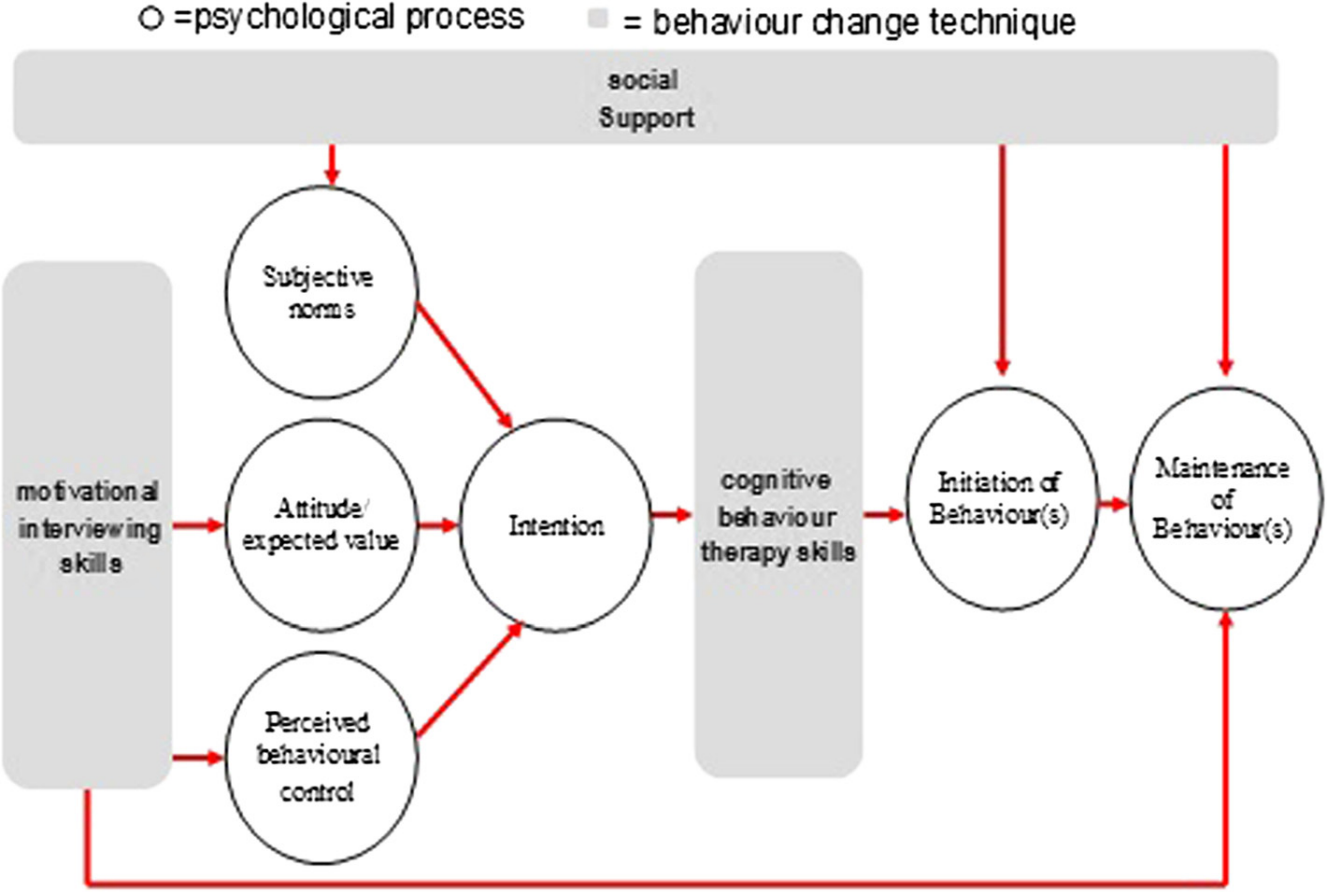
**Intervention map of MOVE-IT for cardiovascular disease risk scores of more than 20%.**

Our intervention will tap into all three constructs using principles and techniques from MI [19], CBT [48] and social cognitive theory [49] (Figure 2). MI will be used to support participants in forming healthy intentions. MI is a collaborative conversation style for strengthening a person’s own motivation, belief and commitment to change. Hobbis and Sutton highlight the gap between translating intention into action and illustrate how CBT can be applied to bridge this gap [48]. For this intervention, techniques from CBT will be used to support the transition from intention to action, and action to maintenance [50]. Identifying and challenging unhelpful thoughts or thinking styles can promote more positive emotions and behaviours [51]. For example, ‘When I get breathless after some exercise (bodily sensation) this means I am going to damage my heart (incorrect cognition)’ or ‘I have eaten one doughnut (behaviour) - I might as well eat the whole bag (all or nothing cognition)’.

Social cognitive theory emphasises the importance of significant others in shaping people’s behaviours. The theory of planned behaviour also highlights this aspect through the ‘subjective norm’ construct. In our intervention, social networks from the participant’s own life and/or group members (in the group arm) will be actively utilised to provide practical and emotional support and opportunities for modeling health behaviours during all phases of the intervention.

##### Intervention development

We will conduct a scoping study to identify manuals published in English in the last five years to improve diet and/or physical activity in the peer-reviewed and grey literature. The aim is to map the quality, contents and cultural diversity of these manuals to inform the content of our intervention. The clinical psychologist will devise the intervention based on this synthesis and on our expertise in developing lifestyle interventions. We will use an iterative process to draft the manual and refine it over two to three cycles. There will be three manual outputs from the trial:

1. A training manual: This will describe the teaching methods, structure and content of the training programme used to train the HLFs.
2. An intervention curriculum: This will describe the outline of each intervention session including key learning points, interactive activities and action planning.
3. A participant workbook: This will include key learning points from each session, action planning worksheets, case studies and a self-monitoring diary for each participant. The participants will also receive a pedometer with guidance on how to use this effectively and access to online, DVD and paper resources around CVD risk.

The programme will consist of 10 sessions, plus an introductory session, spread quarterly over 12 months. The outline of each session is described in Table 1. Each participant allocated to a treatment arm will have a session 0 as an introduction to the intervention, to receive their intervention packs and to become familiar with the HLF. The intensive phase consists of six weekly sessions at the beginning of the first quarter. The first three sessions focus on physical activity and the second three sessions focus on diet. The maintenance phase consists of four sessions delivered at three, six, nine and twelve months. Those randomised to the group arm are encouraged to use the peer learning and peer support environment to facilitate change during the intensive phase and maintenance phase. Each group has typically 10 to 11 participants and sessions last 120 minutes. The intervention will be delivered in local venues such as community halls and health centres. In between sessions and during the follow-up period, participants will be encouraged to communicate with each other (in the group arm) and the HLF (both individual and group). Novel methods and teaching aides are used to supplement the delivery of behaviour change techniques such as visual aids of food labels and/or cue cards, exercise demonstrations, video and/or audio material of patient testimonials, activity-based learning around meal planning and text and/or email reminders. For ease of translation, the key components of the programme have been defined according to Abraham and Michie’s behaviour change technique taxonomy [30]:

**Table 1:** **Proposed programme of sessions**

**Session number**	**Content of session**
	Intensive phase:
Session 0	Focus: Introduce the intervention
	Examples of delivery: structure of the programme, ice breaker, rapport building with HLF, give out pedometers and baseline measures.
Session 1: physical activity	Focus: Increasing routine activity
(Week 1)	Examples of delivery: Elicit views regarding walking more and sitting less and instruction on use of pedometer. Support individual goal setting.
Session 2: physical activity	Focus: Increasing non routine activity
(Week 2)	Examples of delivery: Elicit views on recommended activity levels and reflect on previously enjoyed exercise and its benefits. Provide information/demonstration/leaflets regarding local exercise options. Support individual goal setting.
Session 3: physical activity	Focus: To maintain physical activity changes
(Week 3)	Examples of delivery: Elicit views regarding lapse versus relapse using case studies. Discuss lapse triggers and strategies to manage them. Support individual relapse prevention plans (including implementation intentions).
Session 4: diet	Focus: Increasing health food choices
(Week 4)	Examples of delivery: Elicit views on healthy eating principles. Interactive games regarding healthy snacks. Support individual goal setting.
Session 5: diet	Focus: Decreasing unhealthy food choices
(Week 5)	Elicit views on foods to avoid in excess. Interactive games regarding food labelling and high fat and salt foods. Support individual goal setting.
Session 6: diet	Focus: To maintain dietary changes
(Week 6)	Examples of delivery: Elicit views regarding lapse versus relapse using case studies. Discuss lapse triggers and strategies to manage them. Support individual relapse prevention plans (including implementation intentions).
	Maintenance phase:
Session 7	Focus: Review progress and problem-solve setbacks
(3 months)	Examples of delivery: Highlight positive changes in review session, discuss setbacks and potential ways forward. Support individual relapse prevention plans.
Session 8	Focus: Review progress and problem-solve setbacks
(6 months)	Examples of delivery: Highlight positive changes in review session, discuss setbacks and potential ways forward. Support individual relapse prevention plans.
Session 9	Focus: Review progress and problem-solve setbacks
(9 months)	Examples of delivery: Highlight positive changes in review session, discuss setbacks and potential ways forward. Support individual relapse prevention plans.
Session 10	Focus: Review progress and problem-solve setbacks
(12 months)	Examples of delivery: Highlight positive changes in review session, discuss setbacks and potential ways forward. Support individual relapse prevention plans.

1. Provide information on consequences,
2. Prompt intention formation,
3. Prompt barrier identification,
4. Prompt specific goal setting,
5. Prompt review of behavioural goals,
6. Prompt self-monitoring of behavior,
7. Teach to use prompts or cues,
8. Agree on behavioural contract,
9. Use follow-up prompts,
10. Plan social support or social change,
11. Relapse prevention and
12. Motivational interviewing.

##### Training the healthy lifestyle facilitators

The HLFs are at NHS Band 3 level, employed by King's College Hospital and seconded as appropriate to the CCG. The training programme lasts eight weeks. The teaching is a combination of didactic learning, role plays and feedback, group exercises, reading and case study discussion. The HLFs will use rating scales for self-monitoring of skill progression during role plays. The HLF is deemed ready to administer the intervention when they have achieved a specific competency level [52]. We will adapt existing competency frameworks for behaviour change techniques to this study [53-55]. HLFs are expected to offer sessions between 8 am and 9 pm, enabling flexibility for participants who are in full-time work or have carer roles. Cultural and religious awareness is built in. Regular supervision during the intervention will be provided by the clinical psychologist.

#### Group three: usual care and enhanced motivational interviewing in an individual format

This will have the same components as group two but the components are delivered individually. There will be no opportunity, expectation or guidance for participants to form groups with each other in between sessions. Sessions will last 40 minutes. We have kept the number of sessions the same and reduced the duration of each session to approximately match for attention in the two groups.

### Measurement of outcomes

Interim and outcome assessments are collected by research workers using standardised approaches. All laboratory analyses will be carried out by technicians blind to allocation. The main outcome will be treatment differences between the arms at 24 months, with an interim assessment at 12 months.

#### Primary outcomes

The primary outcomes are change in weight (kg) and physical activity (average number of steps per day) between arms. The same methods of assessment as at baseline will be used.

#### Secondary outcomes

Change in LDL cholesterol and CVD risk score will be assessed. The QRISK2 measurement of CVD risk will be sensitive to changes in weight, cholesterol, blood pressure, diabetes status (HbA1c) and smoking status. The number of fatal and non-fatal CVD events and hospital admissions will be recorded via the Hospital Episodes Statistics database [56].

A secondary physical activity outcome will be the amount of at least moderate physical activity (MVPA) in longer than 10 minute bouts, assessed using the accelerometer output. Changes in dietary habits will be measured via analysis of dietary recall data. Health beliefs and depression at 12 and 24 months will be assessed as measures of mediating processes.

The main perspective for the economic evaluation will be that of the health care system. The EuroQol-5D will be used to generate QALYs for use in the economic analyses [57]. Intervention costs will be calculated, taking into account staff time involved in being trained and delivering interventions, overhead costs and sessions provided. For the group intervention the costs will be apportioned over attendees. Other service use will be measured at baseline, 12 month and 24 month follow-up assessments using an adapted Client Service Receipt Inventory (CSRI) [58]. Costs will be calculated by combining service use data with information on unit costs [59]. Secondary analyses will take into account costs for other agencies and will include lost employment costs.

### Statistical analysis plan

A description of the sample will be presented using means and their standard deviations or counts (proportions). Baseline characteristics of those who were eligible but decline to participate with those who consent to participate and those who dropped out of follow up will be compared with participants who complete the study. Analysis and reporting will be in line with the Consolidated Standards of Reporting Trials (CONSORT) guidelines [60], with primary analyses being on an intention-to-treat basis. The differences in treatment effect between the three arms at 12 and 24 months of this partially nested design will be analysed using mixed-effects models with pre-randomisation values as a covariate [61]. In the linear mixed-model treatment arm, time (as a categorical variable with two levels - 12 and 24 months), the interaction between treatment group and time, borough, ethnicity and the baseline values of the outcome variable are the fixed part of the model. The random parts of the models are GP practice (patients are nested in practices) and therapy group. To account for the partially nested design of the therapy group (only patients within the group therapy are clustered within the same group) an approach which matches the non-parallel data structure will be used [61]. The dependency of the repeated observations of the same subjects at 12 and 24 months and the covariance between the residuals within the lowest level (patients) is to be correlated by using an unstructured covariance pattern model. For the final model the group difference estimates and associated confidence intervals will be reported for 12 (for secondary analyses) and 24 months after randomisation.

The large sample size should ensure that all possible confounding variables are equally distributed between treatment arms. However, in a sensitivity analysis we extend the analyses model of the primary analysis by including baseline variables with substantial imbalance, thought to be important in determining outcome, in the model. The potential baseline variables are age, gender, index of deprivation, education, and marital and smoking status.

The described analyses approach provides valid inferences under the assumption that the missing data mechanism can be ignored (missing at random). Sensitivity of results to missing data will be further assessed by including covariates predictive of missingness in the analyses model, using multiple imputation [62] and exploring the effect of relaxing the missing at random assumption to allow for informative dropout, that is letting missingness also depend on the unobserved value [63].

Health care costs will be compared between the three groups. Given that the data are likely to be skewed, we will use bootstrapping methods to estimate 95% confidence intervals around the mean cost differences. Costs including social care and lost employment will also be compared between the groups. The lost employment costs will be based on days lost from work and average wage rates. However, there is a danger of double-counting between QALYs and lost employment, and therefore the cost-effectiveness analyses will be based on the health service perspective. QALYs will be calculated from the EuroQol-5D administered at baseline, 12 months and 24 months. Area under the curve methods will allow us to calculate the QALY gain over the entire follow-up period. If costs are higher for one group compared to another and QALY gains are greater, we will then construct an incremental cost-effectiveness ratio to show the cost per extra QALY gained. There will be uncertainty around cost and QALY estimates and this will be explored using cost-effectiveness planes generated from 1,000 bootstrapped resamples of the data for each of the three comparisons. Finally, we will generate cost-effectiveness acceptability curves, using the net-benefit approach and bootstrapping, to indicate the probability that any of the three approaches is the most cost-effective for different values placed on a QALY gain. The range of values used will be £0 to 100,000. This includes the threshold that is used by the National Institute for Health and Care Excellence of £20,000 to 30,000. Sensitivity analyses will be carried out around key costs, particularly those for the interventions themselves.

### Qualitative analysis

The overall aim is to identify and describe factors and processes that affect the delivery, receipt and outcome of the study to aid the interpretation and translation of the observed findings. Process data will be analysed before outcome data wherever possible to reduce bias in interpretation. The main themes will be reach, quality (fidelity) and processes of change.

#### Reach

The extent to which the intervention reached out to eligible participants will be assessed by comparing the reasons given by GPs as to why they agree or decline to participate. We will also assess participation and attrition biases; some patients who have declined to participate in the RCT may be willing to give written informed consent to collect baseline data. We will also invite patients who complete less than 50% of sessions to attend a focus group to give feedback on the programme.

#### Quality (fidelity)

We will measure adherence and competence. Adherence is the extent to which the therapist applies the techniques prescribed in the manual and avoids those proscribed. For MI we will use the MI Treatment Integrity (MITI), which counts the number of MI techniques used [52], and for an overall assessment of competency we will adapt emerging competency frameworks [53-55]. There is no consensus as to the number and order of sessions that should be analysed [25]. Considering the potential massive volume of material we will audiotape sections of 25% of all sessions, selected randomly.

#### Processes of change

We will scan copies of self-monitoring worksheets to measure adherence to the intervention. Supervision checklists and interviews with health trainers will be used to assess which behavior change techniques are popular, why and for which lifestyle behaviour. We will administer a detailed process questionnaire at 15 months that requires all randomised participants to discuss, in open-ended and standardised structured questionnaires, which techniques they had found most useful, their appraisal of the techniques and their level of satisfaction with the interventions of their allocated arms. We will include the usual care arm in order to assess the similarity and differences with the intervention as there may be some overlap.

### Ethical issues

The trial has been reviewed by the Dulwich Ethics Committee and has been approved (reference: 12/LO/0917). The Trial Steering Committee (TSC) will provide overall trial supervision supported by the Data Monitoring and Ethics Committee (DMEC). Professor Steve Iliffe, Professor of Primary Care of the Elderly, University College London is the chairperson for the TSC and Professor Helen Weiss, Head of International Epidemiology, London School of Hygiene and Tropical Medicine, is the chairperson for the DMEC. The main ethical consideration is to ensure that the risk of harm to participants is minimised and that they are fully informed of any risks. We will take into account literacy and cultural sensitivities in obtaining informed consent. Other ethical considerations are ensuring that recruitment and informed consent are handled in such a way that potential participants are not put under pressure to take part, and that confidentiality is preserved. All participant data will be stored using a unique study identifier and electronic data will be password protected.

In general, regular physical activity is associated with improved health outcomes and this outweighs the risk of sedentary lifestyles. However, a sudden increase in vigorous physical activity in otherwise sedentary individuals is associated with a higher risk of myocardial infarction and of musculoskeletal injuries, which may be pertinent as we are intervening in a group that is at high risk for CVD. However, one of the components of behaviour change techniques is to deliver the message that physical activity should be increased in a graded manner rather than suddenly. We will be discouraging excessive and/or sudden changes to lifestyles. Weight loss could worsen frailty by accelerating the usual age-related loss of muscle that leads to sarcopenia, but combining weight loss with increased physical activity can actually ameliorate frailty [64]. Importantly, our intervention is based on healthier diets and gradual and sustainable weight loss as opposed to commercial weight loss programmes. We consider risks to be small and minimal due to the exclusion of subjects with existing CVD.

#### Adverse events

An adverse event, which may be classed as serious, is defined as any untoward occurrence during the course of the study which should be reported to the Research Ethics Committee (REC) and TSC within an agreed timeframe. A suspected unexpected serious adverse event (SUSAR) is defined as an untoward occurrence that is related to the intervention and is unexpected. Participants will have the opportunity to report adverse events at 12-month and 24-month study appointments with the researcher, and participants receiving the intervention will be able to report adverse events at any time during the intervention period to the HLF. All serious adverse events and laboratory values will be reviewed by the principal investigator and another co-investigator, and the principal investigator will be responsible for determining causality and reporting any adverse events related to the study to the REC using the National Research Ethics Service guidance.

#### Obtaining informed consent

GP staff will conduct the searches using our guidance and invite potential participants to give permission for the research workers to contact them. Research workers will invite potential participants to meet them in the surgery, and they will be given verbal and written information about the study and at least one week to think about participating. We will invite patients who are eligible but decline participation to give informed consent for the collection of baseline data to assess the generalizability of our findings.

#### Withdrawal and stopping rules

Participants will be free to withdraw from the study at any time. If the participant withdraws from the intervention, they will be asked if they are happy to attend follow-up study appointments and for data to be collected. If they withdraw from the study without consent to follow-up, no further data will be collected.

There are no formal stopping rules. This is due to the fact that the intervention does not ask participants to do anything more than follow usual GP advice regarding diet and physical activity. However, the principal investigator will evaluate the causality of any adverse events and advise withdrawal if necessary.

#### Time period for retention of trial documentation

A copy of patient consent forms will be kept for 12 months after the study has ended. Personal data that are identified by patient name or address will be destroyed three years after the study has ended. Other trial records will be archived for seven years after the trial ends before being destroyed.

## Discussion

This study will compare the effectiveness of enhanced MI interventions, in a group and individual format, with usual care for reducing weight and increasing physical activity in 40 to 74-year-olds with a high risk (≥20%) of developing CVD in the next 10 years. The study will take place across 11 CCGs in South London and will assess changes at 24 months, with an interim assessment at 12 months. The relative effectiveness of group and individual interventions will be assessed; it is predicted that participants in the group intervention will benefit from social support and that this approach will be more cost-effective than the individual format.

The benefits of the study setting include the infrastructure for dissemination of research findings (through the HIN), and that a significant proportion of the South London population is African, Caribbean or South Asian. A limitation is that health inequalities between the north and south of the United Kingdom will not be addressed. However, health inequalities within South London mirror the rest of the United Kingdom, and South London is well-placed in being a geographical setting with a multi-ethnic population across a variably deprived setting, allowing ethnicity and deprivation to be addressed in the study design. Changes in NHS organisation during the course of the study also present challenges, including the transfer of public health responsibilities from the NHS to local government and the change from primary care trusts to CCGs. Poor uptake of the NHS Health Check programme has also prompted changes to recruitment procedures. Variably outdated data on patient records systems may lead to inaccurate estimate of CVD risk and, therefore, large numbers of ineligible participants consented.

The intervention offers the advantage of potentially active ingredients to support maintenance, such as social support within the group intervention and a long-term follow-up period. One challenge is in avoiding selection bias in the study design and ensuring the intervention targets those most at need, as participants who respond to the invite to take part in the study may already be motivated. However, as the participants will have screened at high risk of CVD there is cause for treatment.

During the study, ensuring blinding to treatment allocation may also be an issue. It will not be possible for the participant or HLF to be blind to treatment allocation due to the nature of the intervention. The researcher will be blind to the treatment allocation and will remind the participant about this when carrying out outcome measurements. When unblinding does occur this will be recorded. Objective physical assessments and accelerometer data, as well as a standardised procedure for self-report measures, will limit any researcher bias possible during outcome assessment.

### Trial status

Study appointments started in June 2013 and the intended end date is February 2017. Recruitment of GP surgeries and participants is taking place between June 2013 and January 2015; longer than initially anticipated. Due to poor uptake of the NHS Health Check programme the size of the population to be screened needed to be increased. QRISK2 scores based on patient records database screening need to be confirmed by inviting patients for formal screening. This has led to an almost doubling of the population that needs to be consented, as approximately 40 to 50% of those consented have been ineligible. Therefore, the recruitment target has been increased to 3,000 participants in order to randomise the 1,704 required. The final report will be prepared for June 2017.

## Abbreviations

CBT: cognitive behavioural therapy
CCG: Clinical Commissioning Group
CSRI: Client Service Receipt Inventory
CVD: Cardiovascular disease
DMEC: Data Monitoring and Ethics Committee
GP: General practitioner
GPAQ: Global Physical Activity Questionnaire
HDL: High density lipoprotein
HIN: Health Innovation Network
HLF: Healthy lifestyle facilitator
HTA: Health Technology Assessment
IPAQ: International Physical Activity Questionnaire
LDL: Low density lipoprotein
MI: Motivational interviewing
MITI: Motivational Interviewing Treatment Integrity
MOVE: IT Enhanced motivational interviewing intervention
NHS: National Health Service
NIHR: National Institute for Health Research
QALY: Quality-adjusted life year
RCT: Randomised controlled trial
REC: Research Ethics Committee
SLHIEC: South London Health and Education Cluster
SUSAR: Suspected unexpected serious adverse event
TSC: Trial Steering Committee
